# The role of wobble uridine modifications in +1 translational frameshifting in eukaryotes

**DOI:** 10.1093/nar/gkv832

**Published:** 2015-08-17

**Authors:** Hasan Tükenmez, Hao Xu, Anders Esberg, Anders S. Byström

**Affiliations:** 1 Department of Molecular Biology, Umeå University, Umeå, 901 87, Sweden; 2 Department of Odontology/Cariology, Umeå University, Umeå, 901 87, Sweden

## Abstract

In *Saccharomyces cerevisiae*, 11 out of 42 tRNA species contain 5-methoxycarbonylmethyl-2-thiouridine (mcm^5^s^2^U), 5-methoxycarbonylmethyluridine (mcm^5^U), 5-carbamoylmethyluridine (ncm^5^U) or 5-carbamoylmethyl-2′-O-methyluridine (ncm^5^Um) nucleosides in the anticodon at the wobble position (U_34_). Earlier we showed that mutants unable to form the side chain at position 5 (ncm^5^ or mcm^5^) or lacking sulphur at position 2 (s^2^) of U_34_ result in pleiotropic phenotypes, which are all suppressed by overexpression of hypomodified tRNAs. This observation suggests that the observed phenotypes are due to inefficient reading of cognate codons or an increased frameshifting. The latter may be caused by a ternary complex (aminoacyl-tRNA*eEF1A*GTP) with a modification deficient tRNA inefficiently being accepted to the ribosomal A-site and thereby allowing an increased peptidyl-tRNA slippage and thus a frameshift error. In this study, we have investigated the role of wobble uridine modifications in reading frame maintenance, using either the *Renilla*/*Firefly* luciferase bicistronic reporter system or a modified Ty1 frameshifting site in a *HIS4A::lacZ* reporter system. We here show that the presence of mcm^5^ and s^2^ side groups at wobble uridines are important for reading frame maintenance and thus the aforementioned mutant phenotypes might partly be due to frameshift errors.

## INTRODUCTION

Transfer of genetic information from mRNA into proteins is the most energy consuming process in the cell and the translation machinery needs to decode mRNAs with high efficiency and fidelity (1). Even though the translational machinery transfers the information in mRNA into protein with high fidelity, errors occur at a low frequency. Missense errors are in most cases not harmful to the function of a protein, since such errors alter only one single amino acid, which will not interfere with the function or stability of the protein if they occur in non-critical positions. In contrast, processivity errors, like frameshift errors, are detrimental, since they completely change the amino acid sequence downstream of the frameshift site. Moreover, following such an error, the ribosome frequently encounters a stop codon in the new reading frame resulting in premature termination of translation. Accordingly, the frequency of frameshift errors is about 10-fold lower than the frequency of missense errors (1,2).

There are many examples where alterations in the tRNA structure, e.g. lack of a modified nucleoside, will affect the fidelity of reading frame maintenance (3,4). In bacteria, modified nucleosides of different chemical structures, present in different positions, and in different species of the tRNA all prevent frameshifts errors (5,6). In eukaryotes, both wyosin (yW) and queosine (Q) in rabbit reticulocytes as well as other modified nucleosides present in the anticodon loop of eukaryotic tRNAs are important to maintain the reading frame (7,8). Synthesis of yW in yeast tRNA occurs in several steps and whereas fully modified yW has a low frequency of frameshifting, presence of any of the various intermediates in the synthesis of yW all increase frameshifting (9). Also, lack of either cyclic N6-threonylcarbamoyladenosine (ct^6^A) at position 37 or pseudouridine (Ψ) at position 38 and 39 in yeast tRNA increases +1 frameshifting (10–13). Relevant for this study, the modified wobble nucleoside 5-methylaminomethyl-2-thiouridine (mnm^5^s^2^U_34_) present in bacterial tRNA specific for Gln, Lys and Glu, is important for proper reading frame maintenance (The wobble nucleoside is in position 34 of the tRNA and we denote such a nucleoside as N_34_ where N is any nucleoside.) (6,14–17). Apparently, modification status both in bacteria and in eukaryotes is important for a proper reading frame maintenance (3,4).

**Figure 1.**
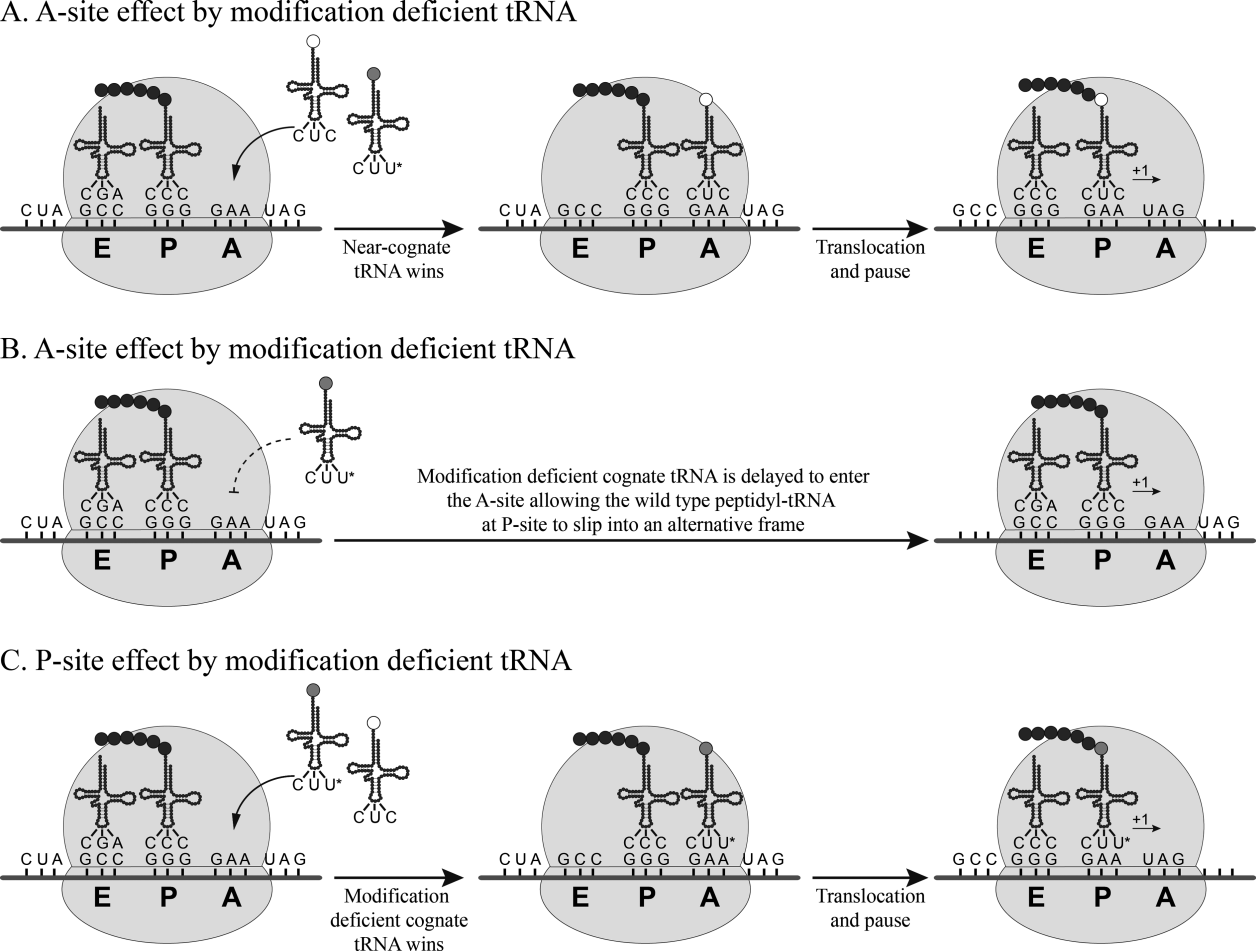
Dual-error frameshifting model. Modification deficient tRNAs can induce frameshifting by either an A- or a P-site effect, or a combination thereof. (**A**) Lack of wobble uridine modification reduces the efficiency of the ternary complex (aminoacyl-tRNA*eEF1A*GTP, here shorten as aminoacyl-tRNA) to be accepted to the A-site, allowing a near cognate aminoacyl-tRNA to be accepted in the A-site. After translocation to the P-site, the near cognate tRNA slips into an alternative reading frame, as it does not perfectly fit in the P-site. (**B**) Lack of wobble uridine modification reduces the efficiency of the cognate aminoacyl-tRNA to be accepted to the A-site, which induces a pause that allows the tRNA in the P-site to frameshift. (**C**) The hypomodified aminoacyl-tRNA is able to enter the A-site and translocate to the P-site where it then slips into an alternative reading frame due to a reduced ribosomal grip.

A peptidyl-tRNA slippage model of how tRNA modification deficiency may induce frameshifting errors is well established (3,4,6,18–24). According to this model (Figure 1) modification deficient aminoacyl-tRNAs present in a ternary complex, i.e. aminoacyl-tRNA*eEF1A*GTP (here shorten as aminoacyl-tRNA) induces frameshifts either by causing an A- or a P-site effect, or a combination thereof. Lack of modification causes a defect in the cognate aminoacyl-tRNA selection step (we denote such an error as an A-site effect by modification deficiency), allowing a ternary complex with a near cognate wild type aminoacyl-tRNA instead of a cognate aminoacyl-tRNA to be accepted in the A-site. After translocation to the P-site, the fit of the near cognate peptidyl-tRNA is not optimal why it slips one nucleotide forward (+1 frameshift) (Figure 1A). Alternatively, lack of a modified nucleoside reduces the efficiency by which a cognate aminoacyl-tRNA is accepted to the A-site, which induces a ribosomal pause allowing the wild type peptidyl-tRNA to slip forward one nucleotide (denoted an A-site effect by modification deficiency, Figure 1B). When frameshifting is caused by a P-site effect, the hypomodified tRNA is efficiently accepted to the A-site, translocates to the P-site where its fit is not optimal why it slips into an alternative reading frame due to a reduced ribosomal grip (P-site effect by modification deficiency, Figure 1C) (3,6,20,21,23). Thus, in some cases, the modification deficiency reduces the rate of selection of the aminoacyl-tRNA (A-site effect) but also lack of the modification reduces the ribosomal grip in the P-site (P-site effect). Note, in all cases explained above, the error in reading frame maintenance is due to a peptidyl-tRNA slippage.

**Figure 2.**
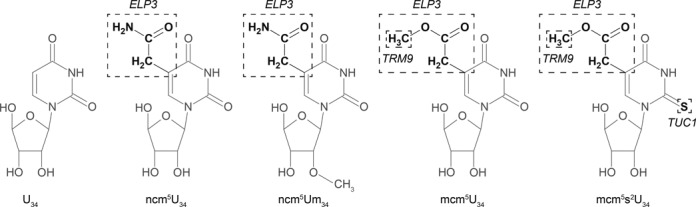
Chemical structures of uridine, 5-carbamoylmethyluridine (ncm^5^U), 5-carbamoylmethyl-2′-O-methyluridine (ncm^5^Um), 5-methoxycarbonylmethyluridine (mcm^5^U) and 5-methoxycarbonylmethyl-2-thiouridine (mcm^5^s^2^U) nucleosides. Each dotted box indicates the side group that is removed by mutating the indicated gene.

**Figure 3.**
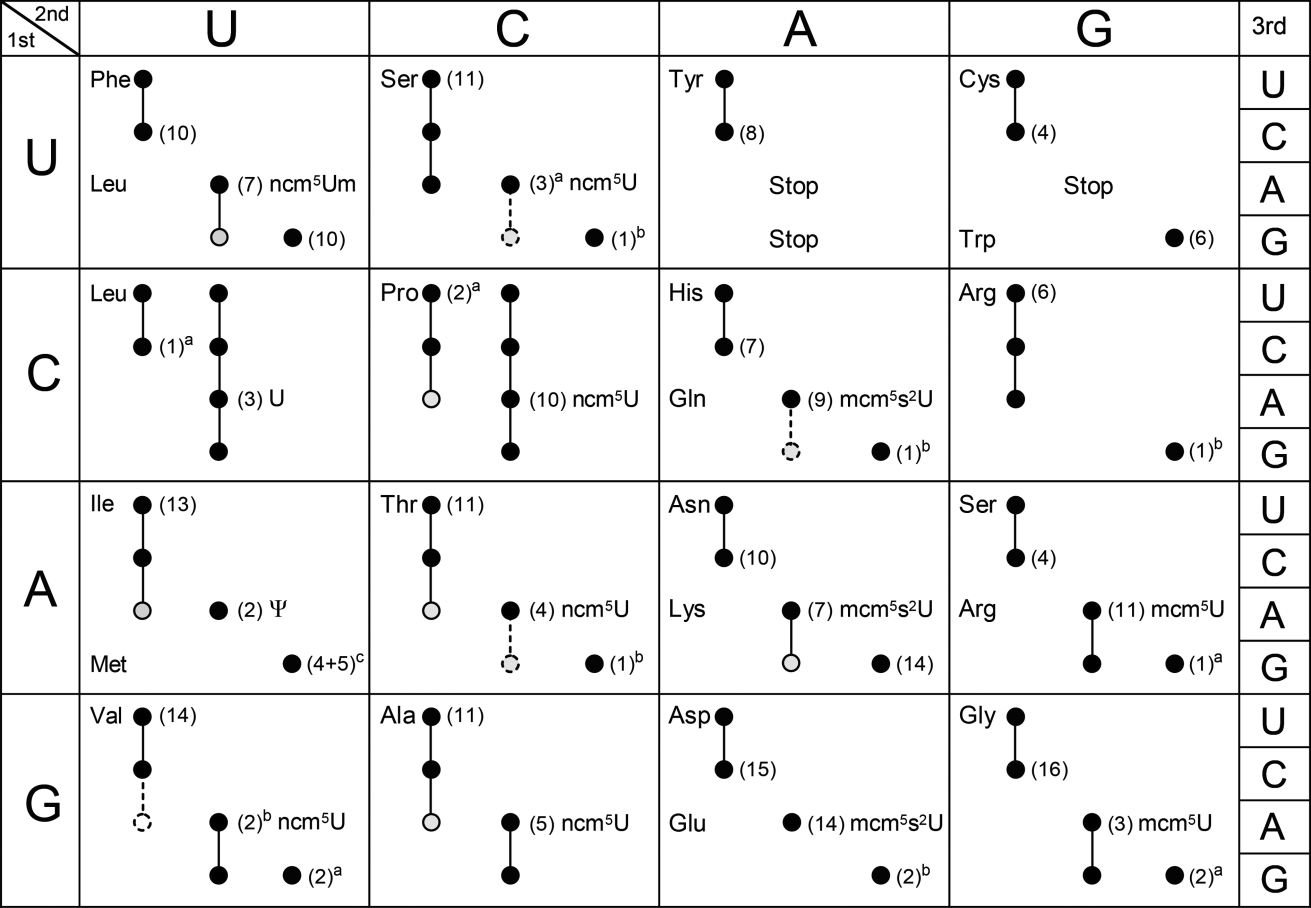
The genetic code and decoding abilities of individual tRNA species. Circles and connecting lines indicate codons read by the same tRNA isoacceptor. Gray circles connected with a dashed line indicate that the tRNA species reads the codon only when it is overexpressed. The empty dashed circle for ${\rm tRNA}_{{\rm IAC}}^{{\rm Val}}$ is shown only to indicate that this inosine containing tRNA species does not efficiently read the GUA codon. The nucleoside at the wobble position is given for the 13 wobble uridine containing tRNA species. Black and gray circles represent decoding abilities predicted by the wobble hypothesis, the revised wobble rules and the distribution of tRNA species. A gray circle indicates that the tRNA species is less likely to read the codon. The number of genes coding for a tRNA species is indicated next to the circle for the complementary codon. ^a^ The gene(s) encoding the tRNA is nonessential. ^b^ The gene(s) encoding the tRNA is essential. ^c^ Four genes code for ${\rm tRNA}_{\rm i}^{{\rm Met}}$and five for ${\rm tRNA}_{\rm m}^{{\rm Met}}$. Copyright © American Society for Microbiology, [Molecular and Cellular Biology, 28, 2008, 3301–3312 and doi:10.1128/MCB.01542–07](26).

Modifications of uridines in the wobble position of tRNAs are frequent in all three domains of life. In *Saccharomyces cerevisiae*, there are 11 tRNA species having four related modified uridine nucleotides at wobble position (25–32). These modified nucleosides are 5-carbamoylmethyluridine (ncm^5^U_34_) present in five (26,27,32), 5-carbamoylmethyl-2′-O-methyluridine (ncm^5^U_34_m) present in one (25), 5-methoxycarbonylmethyluridine (mcm^5^U_34_) present in two (29,30) and 5-methoxycarbonylmethyl-2-thiouridine (mcm^5^s^2^U_34_) present in three tRNA species (Figures 2 and 3) (28,30,31).

The first step in the synthesis of the mcm^5^ and ncm^5^ groups of the uridine modifications mentioned above requires the six-subunit Elongator complex and its seven associated proteins (Reviewed in Karlsborn et al. (33)). Mutations in any of the corresponding genes result in deficiency of these xm^5^-uridine modifications without affecting stability or aminoacylation of tRNA (26). These mutants also show strong pleiotropic phenotypes, such as defects in growth, transcription, chromatin remodelling, DNA repair and secretion (Reviewed in Karlsborn et al. (33)). All these phenotypes, except lack of xm^5^ side chains, are suppressed by overexpression of hypomodified tRNAs specific for Gln, Lys and Glu that in a wild type contains mcm^5^s^2^U_34_ (34,35). It was concluded that lack of this wobble nucleoside reduces the efficiency to recognize the cognate codons for these tRNAs, which is compensated by an increased concentration of the modification deficient tRNA. Thus, the many different phenotypes of Elongator mutants are due to reduced efficiency in translating some key mRNAs encoding proteins important for manifesting a correct phenotype.

In bacteria, modified wobble uridines are important to prevent +1 frameshifting (6,36). In eukaryotes, only a limited study has been done, which focused on the influence of the esterified methyl group of mcm^5^U_34_ in reading frame maintenance (37). However, no specific conclusion was made where the frameshift errors occur, since the frameshift window used was very large. Therefore, no extensive information of the role of modified wobble uridines in reading frame maintenance is available for eukaryotic tRNA. It was therefore important to investigate whether or not lack of the xm^5^U or mcm^5^s^2^U modifications are crucial for reading frame maintenance. Here, we show that presence of xm^5^- (x, any substitution) or s^2^ side groups at wobble uridines in yeast is pivotal in maintaining the translational reading frame.

## MATERIALS AND METHODS

### Strains, media and genetic procedures

The source and genotypes of yeast strains used in this study are listed in Table 1. *E. coli* strain used was *DH5α* (Bethesda Research Laboratories). Yeast transformation (38), media and genetic procedures have been described previously (39).

**Table 1.** Yeast strains used in this study

Strains	Genotype	Source
W303–1A	*MATa leu2–3,112 trp1–1 can1–100 ura3–1 ade2–1 his3–11,15*	(52)
UMY3269	*MATa leu2–3,112 trp1–1 can1–100 ura3–1 ade2–1 his3–11,15 elp3::KanMX4*	(30)
UMY3164	*MATa leu2–3,112 trp1–1 can1–100 ura3–1 ade2–1 his3–11,15 tuc1::TRP1*	(48)
UMY3267	*MATa leu2–3,112 trp1–1 can1–100 ura3–1 ade2–1 his3–11,15 trm9::KanMX4*	(53)

### Plasmid constructions

Plasmid pJD375 contains a *Renilla*/*Firefly* luciferase bicistronic reporter system (40). To introduce various frameshifting windows between the luciferase genes, a BamHI-XhoI fragment from plasmid pJD375 containing the *Firefly* luciferase gene was cloned into corresponding sites of YCp50, generating plasmid YCp50-*Firefly*. Two complementary oligonucleotides carrying various frameshifting windows (see Supplementary Table S1) were annealed into the BamHI and SacI sites of YCp50-*Firefly*. The newly constructed plasmids were digested with restriction enzymes (BamHI and XhoI) and fragments containing the frameshifting sites linked to the *Firefly* luciferase gene were cloned back into the corresponding sites of pJD375 restoring the bicistronic reporter system with the frameshifting window.

**Figure 4.**
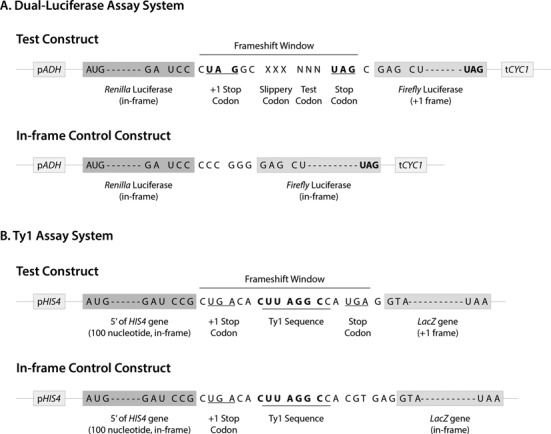
(**A**) Schematic drawing of the dual-luciferase assay system. Transcription of the genes encoding the *Renilla-* and *Firefly-*luciferase is under the *ADH1* promoter and terminated by *CYC1* terminator. Frameshift sites were cloned between the luciferase genes and expression of the *Firefly* luciferase gene requires +1 frameshifting. The frameshifting site is as follows: XXX-slippery site, NNN-assay codon and UAG-stop codon (all in-frame). An upstream stop codon (UAG) was placed in the +1 frame to eliminate frameshifting events occurring before the assay site. The frame of the different luciferase genes is indicated. In the in-frame control construct, *Renilla-* and *Firefly-*luciferase genes are in-frame. (**B**) Schematic drawing of the Ty1 assay system. Transcription from *HIS4* promoter generates a transcript containing the first 100 nucleotides of the *HIS4* gene in the in-frame and the *lacZ* gene of *Escherichia coli* in the +1 frame. Expression of the *lacZ* gene is dependent on a +1 ribosomal frameshift event taking place within Ty1 sequence. An upstream stop codon (UGA) was placed in the +1 reading frame to eliminate frameshifting events occurring before the assay site. In the in-frame control construct, the first 100 nucleotides of the *HIS4* gene and *lacZ* gene are in-frame.

Plasmids pMB38–9mer (FF and WT) contain a *HIS4A::lacZ* reporter cassette. In pMB38–9merFF (in-frame control construct), the *lacZ* gene is in 0 frame, while in pMB38–9merWT (test construct), the *lacZ* gene is in +1 frame (Figure 4B) (41). These plasmids were used as templates for PCR oligonucleotide directed mutagenesis to alter the Ty1 sequence (CTT-AGG-C) (Figure 4B and Supplementary Table S2).

For the overexpression of the Lys-tRNA encoded by the *tK(UUU)L* gene, we first introduced SphI and NheI restriction sites to plasmids pMB38–9mer (FF and WT) carrying the ‘CUU-AAA-C’ sequence by PCR oligonucleotide directed mutagenesis. Oligonucleotides used were 5′-GGTGTCGGGGCGCATGCATGACCCAGTCAC-3′ and 5′-AGAGTGCACCATATGCGGTGTGAGCTAGCGCACAGATGCG-3′. The *tK(UUU)L* gene was amplified from strain UMY2067 by using oligonucleotides 5′ AAAAGCATGCCGGTAGAGTCTCTT-CTTGGTC-3′ and 5′ AAAAGCTAGCCGGTA-AGAGAGAAACCTCCA-3′ and cloned between SphI and NheI sites of these plasmids.

### Dual-luciferase assays

Three individual transformants of each dual luciferase assay construct (biological replicates) were grown at 30°C in synthetic complete (SC)-Ura medium to an optical density at 600 nm (OD_600_) of 0.5. For each transformant triplicate samples (technical replicates) of 10 μl cells were collected and kept at -80°C. The luciferase assays were performed according to the instructions of Dual-Luciferase Reporter Assay System (Promega, Catalog No. E1960). The luciferase activities were determined in a white 96-well plate (Thermo Scientific, #436111) using a TECAN infinite 200 luminometer. The levels of +1 frameshifting (%) were determined by normalization of each biological test replicate with the average of the three biological replicates of the in-frame control. Each value of the biological replicates was determined by taking the median of the three technical replicates. The significant differences between wild type and mutant were determined by two-tail *t*-test.

### β-galactosidase assays

Three transformants of each Ty1 assay construct (biological replicates) were grown in SC-Ura to OD_600_≈0.5 and 20 OD_600_-units were collected and kept at -20°C. For each transformant, β-galactosidase measurements were done three times (technical replicates). β-galactosidase activities were determined as described previously (39). Values of the biological replicates were determined by taking the median of the technical replicates. The levels of +1 frameshifting (%) were determined by normalization of each biological test replicate with the average of the three biological replicates of the in-frame control. The significant differences between wild type and mutant were determined by two-tail *t*-test.

## RESULTS AND DISCUSSION

### Assay system

To analyze the role of wobble uridine modifications ncm^5^U, ncm^5^Um, mcm^5^U or mcm^5^s^2^U in reading frame maintenance, we used defined yeast mutants unable to form the s^2^ group (*tuc1Δ*; also denoted as *ncs6Δ*), the ncm^5^ or mcm^5^ groups (*elp3Δ)* or the esterified methyl group (*trm9Δ*) of the mcm^5^ side chain.

The ribosomal +1 frameshifting assay system used contains a *Renilla* luciferase (*R-luc*)/ *Firefly* luciferase (*F-luc*) bicistronic reporter system (Figure 4A) (see Material and Methods) (40). This bicistronic mRNA synthesizes a two domain protein with the indicated enzymatic activities. To analyze a +1 frameshift event a sequence is introduced between these two cistrons in such a way that translation of *R-luc* is in the 0 frame and the *F-luc* is in the +1 frame (Figure 4A). To obtain *F-luc* activity the ribosome must shift into the +1 frame before entering the *F-luc* gene. The inserted sequence between the *R-luc* and the *F-luc* reporter genes consists of a slippery codon (XXX) at which the peptidyl-tRNA will slip, the codon to be assayed for A-site selection (NNN), followed by a stop codon in zero frame (UAG) (Figure 4A). To terminate all ribosomes that have accidentally slipped into the +1 frame upstream the slippery codon, a stop codon was inserted in the +1 frame just a few nucleotides upstream the slippery codon (See Figure 4). Thus, to obtain *F-luc* activity a +1 frameshift must occur at the +1 frameshift sequence upstream of the stop codon in the zero frame. This construct results in a very short frameshifting window between the upstream stop codon in the +1 frame and the downstream in-frame stop codon. The slippery codon is determined individually for different assay sites in order to optimize the slippage of the peptidyl tRNA at the P-site. We chose UUU, CCC or GGG codons as the slippery codons (Supplementary Figure S1 and Table 2 and Supplementary Table S1). Codon UUU is decoded by ${\rm tRNA}_{{\rm GAA}}^{{\rm Phe}}$, which has the wobble nucleoside Gm_34_ (42), and its structure is not affected by the *elp3, tuc1* or *trm9* mutations. Codon CCC is read by the I_34_ (inosine) containing ${\rm tRNA}_{{\rm IGG}}^{{\rm Pro}}$ and the ncm^5^U_34_ containing ${\rm tRNA}_{{\rm ncm}^5 {\rm UGG}}^{{\rm Pro}}$ and the slippery codon GGG is read by the mcm^5^U_34_ containing ${\rm tRNA}_{{\rm mcm}^5 {\rm UCC}}^{{\rm Gly}}$ and the C_34_ containing ${\rm tRNA}_{{\rm CCC}}^{{\rm Gly}}$ (Figure 3) (26,30,43). Note that the structures of the ncm^5^U containing ${\rm tRNA}_{{\rm ncm}^5 {\rm UGG}}^{{\rm Pro}}$ reading the slippery codon CCC and the mcm^5^U_34_ containing ${\rm tRNA}_{{\rm mcm}^5 {\rm UCC}}^{{\rm Gly}}$ reading the slippery codon GGG are affected by the *elp3* mutation and might therefore obscure the monitoring of an A-site effect at these test codons. These issues will be addressed below. As a control, we used a construct carrying the *R-luc* and *F-luc* genes in-frame. By dividing the ratio of *F-luc*/*R-luc* activities generated from the frameshifting construct with the ratio of activities from the *F-luc*/*R-luc* in-frame control, the level of frameshifting was revealed. Using these reporter systems, the level of frameshifting for specific tRNA isoacceptors was investigated in the presence or absence of s^2^, ncm^5^, mcm^5^ groups or the esterified methyl group of the mcm^5^ side chain at U_34_.

**Table 2.**
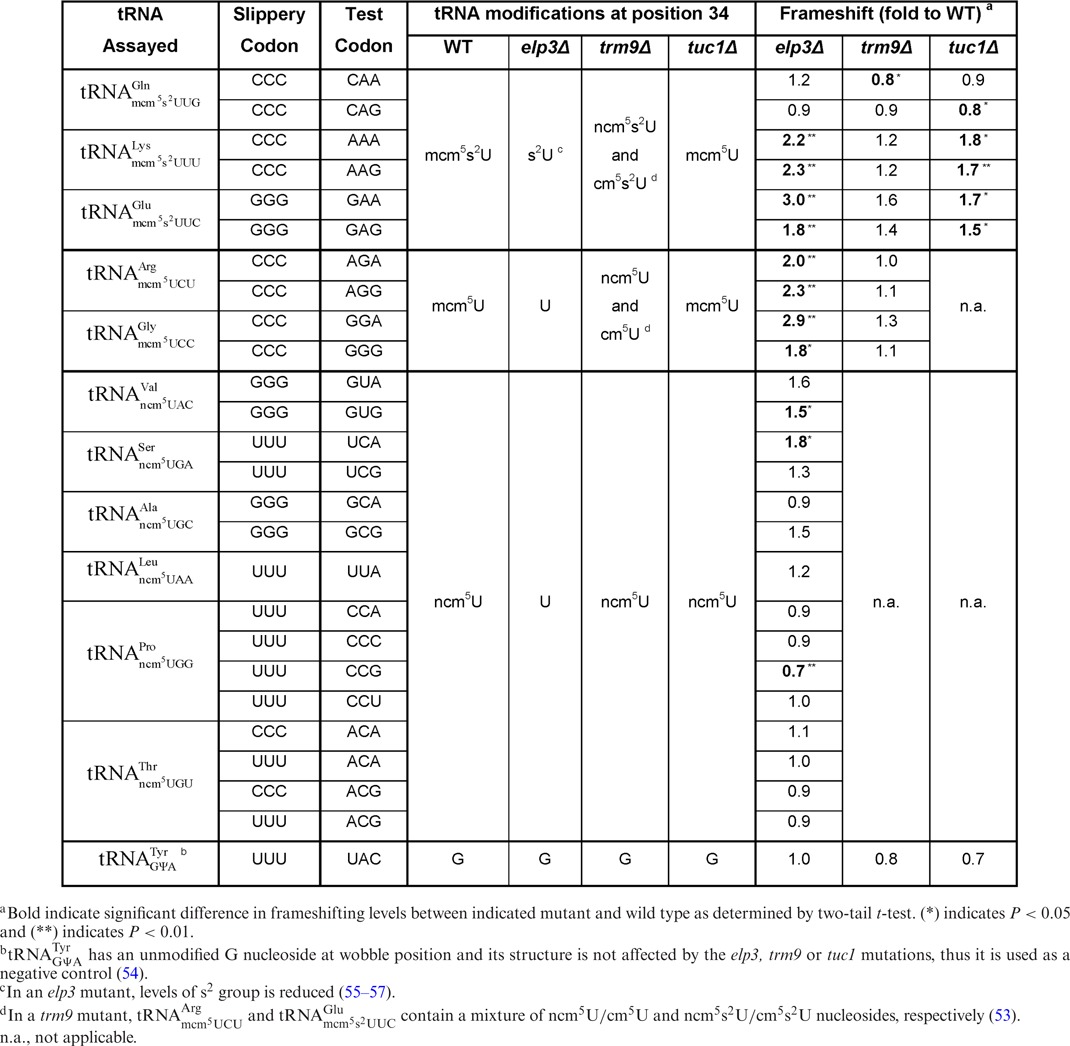
Influence of tRNA modifications mcm^5^s^2^U_34_, mcm^5^U_34_, ncm^5^U_34_ and ncm^5^U_34m_ on reading frame maintenance based on data using the *Renilla/Firefly* luciferase bicistronic reporter system

In the bacterial system, modification deficiency of aminoacyl-tRNA in the ternary complex causes in most cases a slow entry of it to the A-site and thereby induces a peptidyl-tRNA slippage (Figure 1A and B) (6). Therefore, we suspected that in the cases below where we observed an effect on the frequency of frameshifting in the modification deficient mutants, it would primarily be due to an A-site effect, i.e. slow entry of the ternary complex containing aminoacyl-tRNA cognate to the test codon allowing a peptidyl-tRNA interacting with the slippery codon XXX to slip (Figure 1A and B). In the constructs used, all have a UAG stop codon just after the test codon NNN (i.e. the sequence is in zero frame -XXX-NNN-UAG). Translational termination in yeast is controlled by two interacting protein chain release factors, eRF1 and eRF3. Whereas eRF1 recognizes all three stop codons, binds to ribosomal A-site, and promotes hydrolysis of the P-site located peptidyl-tRNA, eRF3 stimulates the termination activity of eRF1 (Reviewed in Kisselev and Buckingham (44)). A poor eRF1 binding to the UAG in the A-site may induce slippage by the modification deficient peptidyl-tRNA from cognate NNN codon to NN-U codon. Therefore, the +1 frameshifting observed using the luciferase assay may be caused by either an A-site or a P-site effect or both. Note that, in all these cases the error occurs in the P-site (either by the tRNA reading the slippery codon or a tRNA cognate to the test codon). Although the luciferase system used by us is unable to distinguish between an A- or a P-site effect caused by modification deficiency, it is still a valuable method to address whether or not modification is important for maintaining the reading frame. To address specifically if modification deficiency induces an A- or a P-site effect, we used the Ty1 system, which is explained below.

### Role of xm^5^U or mcm^5^s^2^U nucleosides in reading frame maintenance

In yeast, there are 11 tRNA species having mcm^5^s^2^U_34_, mcm^5^U_34_, ncm^5^U_34_ or ncm^5^U_34_m nucleosides at wobble position (Figures 2 and 3). The role of these modified uridines was analyzed for ribosomal +1 frameshifting using the *Renilla/Firefly* luciferase bicistronic reporter system described in the previous section.

In an *elp3* mutant these tRNA species are missing the ncm^5^ and mcm^5^ groups at wobble position (U_34_) (26,45). The role of the ncm^5^ and mcm^5^ groups present in these tRNAs in reading frame maintenance was investigated in a wild type and in an *elp3* mutant strain using cognate or near cognate codons as test codons. Lack of the mcm^5^ side chain in ${\rm tRNA}_{{\rm mcm}^5 {\rm UCU}}^{{\rm Arg}}$, ${\rm tRNA}_{{\rm mcm}^5 {\rm UCC}}^{{\rm Gly}}$, ${\rm tRNA}_{{\rm mcm}^5 {\rm s}^2 {\rm UUU}}^{{\rm Lys}}$ and ${\rm tRNA}_{{\rm mcm}^5 {\rm s}^2 {\rm UUC}}^{{\rm Glu}}$ resulted in significantly higher levels of +1 frameshifting with either A-ending cognate or G-ending near cognate codons (Table 2 and Supplementary Figure S1). However, absence of the mcm^5^ group in ${\rm tRNA}_{{\rm mcm}^5 {\rm s}^2 {\rm UUG}}^{{\rm Gln}}$ did not have any significant effect on reading frame maintenance for the Gln codons CAA or CAG (Table 2). Lack of the ncm^5^ group of U_34_ in ${\rm tRNA}_{{\rm ncm}^5 {\rm UAC}}^{{\rm Val}}$ and ${\rm tRNA}_{{\rm ncm}^{\rm 5} {\rm UGA}}^{{\rm Ser}}$resulted in an increased level of +1 frameshifting with near cognate Val codon GUG or cognate Ser codon UCA. In contrast, absence of the ncm^5^ group of U_34_ in ${\rm tRNA}_{{\rm ncm}^{\rm 5} {\rm UGG}}^{{\rm Pro}}$ resulted in a decreased level of +1 frameshifting with near cognate Pro codon CCG. Lack of the ncm^5^ group of U_34_ of the remaining tRNAs did not cause a significant difference in levels of +1 frameshifting (Table 2 and Supplementary Figure S1). We conclude that in the xm^5^U and mcm^5^s^2^U tRNA isoacceptors, the mcm^5^ group plays a more vital role than the ncm^5^ group in reading frame maintenance.

Codon CCC that can be read by ncm^5^U_34_ containing ${\rm tRNA}_{{\rm ncm}^{\rm 5} {\rm UGG}}^{{\rm Pro}}$ is used as a slippery codon upstream next to Gln-, Lys, Arg-, Gly- and Thr- test codons (Table 2 and Supplementary Figure S1). Thus, the ncm^5^U_34_ present in the potential peptidyl-Pro-tRNA might influence the ribosomal grip in the P-site and thus influence the slippage. To test directly the influence of the ncm^5^ group in Pro-tRNA in peptidyl-Pro-tRNA slippage, we used a construct -UUU-CCC-UAG- in the luciferase system. The stop codon UAG is in the zero frame just after the Pro codon CCC. Since eukaryotic release factor 1 (eRF1) acts in the A-site (Reviewed in Kisselev and Buckingham (44)) a possible +1 frameshift by Pro-tRNA lacking ncm^5^U would occur in the P-site. However, no significant +1 frameshifting was observed when the CCC codon was just upstream the stop codon and thus in the P-site (Table 2 and Supplementary Figure S1). We conclude that the ncm^5^ group in Pro-tRNA does not increase peptidyl-tRNA slippage at the slippery codon CCC.

The slippery codon GGG is decoded by both C_34_ containing ${\rm tRNA}_{{\rm CCC}}^{{\rm Gly}}$ and mcm^5^U_34_ containing ${\rm tRNA}_{{\rm mcm}^{\rm 5} {\rm UCC}}^{{\rm Gly}}$. The structure of the latter tRNA is affected by the *elp3* mutation and this tRNA reads the GGG codon very inefficiently compared to the cognate ${\rm tRNA}_{{\rm CCC}}^{{\rm Gly}}$ (26). Therefore, in the *elp3* mutant it is not likely that ${\rm tRNA}_{{\rm mcm}^{\rm 5} {\rm UCC}}^{{\rm Gly}}$ lacking the mcm^5^ side chain at U_34_ will out-compete the efficiently decoding cognate ${\rm tRNA}_{{\rm CCC}}^{{\rm Gly}}$ at GGG codons. Consequently, the observed +1 frameshifting for ${\rm tRNA}_{{\rm mcm}^{\rm 5} {\rm s}^{\rm 2} {\rm UUC}}^{{\rm Glu}}$ and ${\rm tRNA}_{{\rm ncm}^{\rm 5} {\rm UAC}}^{{\rm Val}}$ (Table 2) is most likely not caused by slippage of an unmodified peptidyl-${\rm tRNA}_{{\rm mcm}^{\rm 5} {\rm UCC}}^{{\rm Gly}}$ but rather a poor A-site entry by ${\rm tRNA}_{{\rm mcm}^{\rm 5} {\rm s}^{\rm 2} {\rm UUC}}^{{\rm Glu}}$ and ${\rm tRNA}_{{\rm ncm}^{\rm 5} {\rm UAC}}^{{\rm Val}}$, respectively.

The formation of the esterified methyl group of mcm^5^U, which is the last step in the synthesis of the mcm^5^ side chain, is catalyzed by the dimeric Trm9/Trm112 protein complex (46,47).

The influence of the esterified methyl group of the mcm^5^U_34_ and mcm^5^s^2^U_34_ nucleoside in reading frame maintenance was investigated in a wild type and in a *trm9* mutant strain using cognate or near cognate codons as test codons (Table 2). Lack of the esterified methyl group of the mcm^5^ side chain of U_34_ in ${\rm tRNA}_{{\rm mcm}^{\rm 5} {\rm s}^{\rm 2} {\rm UUG}}^{{\rm Gln}}$ resulted in significantly decreased +1 frameshifting at the Gln codon CAA (Table 2 and Supplementary Figure S1). There were no significant differences in the levels of frameshifting between wild type and *trm9* mutant in the remaining test constructs (Table 2 and Supplementary Figure S1). We conclude that presence or absence of the esterified methyl group in the mcm^5^ side chain only seem to play a minor role in +1 frameshifting.

In a *tuc1* mutant, the s^2^ group of mcm^5^s^2^U at the wobble position (U_34_) is absent in ${\rm tRNA}_{{\rm mcm}^{\rm 5} {\rm s}^{\rm 2} {\rm UUG}}^{{\rm Gln}}$, ${\rm tRNA}_{{\rm mcm}^{\rm 5} {\rm s}^{\rm 2} {\rm UUU}}^{{\rm Lys}}$ and ${\rm tRNA}_{{\rm mcm}^{\rm 5} {\rm s}^{\rm 2} {\rm UUC}}^{{\rm Glu}}$ (48). The role of the s^2^ group present in these tRNAs in reading frame maintenance was investigated in a wild type and a *tuc1* mutant strain using cognate or near cognate codons as test codons (Table 2). Absence of the s^2^ group in ${\rm tRNA}_{{\rm mcm}^{\rm 5} {\rm s}^{\rm 2} {\rm UUU}}^{{\rm Lys}}$ and ${\rm tRNA}_{{\rm mcm}^{\rm 5} {\rm s}^{\rm 2} {\rm UUC}}^{{\rm Glu}}$ resulted in significantly higher levels of +1 frameshifting with either A-ending cognate or G-ending near cognate codons (Table 2 and Supplementary Figure S1). However, lack of the s^2^ group in ${\rm tRNA}_{{\rm mcm}^{\rm 5} {\rm s}^{\rm 2} {\rm UUG}}^{{\rm Gln}}$ resulted in significantly decreased +1 frameshifting with the near cognate CAG (Table 2 and Supplementary Figure S1).

In the cases stated above, Gln- and Pro-tRNAs showed reduced levels of +1 frameshifting due to lack of esterified methyl, s^2^ or ncm^5^ groups (Table 2). This reduced level of frameshifting might be surprising but similar observations were noted earlier. In bacteria the Gln-, Lys- and Glu-tRNA contain as wobble nucleoside the mnm^5^s^2^U, which is structurally related to the mcm^5^s^2^U present in the corresponding yeast tRNAs. Lack of either the mnm^5^ side chain or the sulphur at position 2 reduced frameshifting similarly as noted by us for the two aforementioned cases (15,16). Although these results seems counterintuitively strange, one has to remember that the structure of the different tRNA species is optimized and in fact has evolved to have similar decoding activity, which is obtained partly due to modification of it (49). Therefore, a modification may improve the activity of one tRNA whereas it might reduce the activity of another tRNA species (See discussion of this issue in Björk and Hagervall (4)). From such considerations, one would expect that when measuring a specific activity of a tRNA, like influencing reading frame maintenance, a modification might improve or reduce the fidelity of it.

### The frameshifting error occurs by peptidyl-tRNA slippage

A key feature of the peptidyl-tRNA slippage model is that the error in reading frame maintenance, induced either by an A- or a P-site effect due to modification deficient tRNA, occurs in the P-site by peptidyl-tRNA slippage. There are two ways to establish if the frameshift errors occur in the ribosomal A- or P-site. Either one determines the amino acid sequence of the frameshift peptide covering the frameshift window or by overexpressing the tRNA cognate to the A-site codon. In the latter case, if the frameshift error occurs due to an A-site effect, such overexpression would decrease the frameshift error, since it reduces the ribosomal pause and thereby reduces the ability of the peptidyl-tRNA to slip forward. We chose the latter method, since this approach is relevant for this study, as such a treatment also suppresses all the pleiotropic phenotypes induced by a mutation in, e.g. the *ELP3* gene. Thus, the strong pleiotropic phenotypes observed in an *elp3* mutant might be due, at least partly, to errors in reading frame maintenance of some key mRNAs.

As stated in the description of the assay system, the dual-luciferase assay system is not designed to clarify the difference between an A- or a P-site effect caused by modification deficiency, we decided to use Ty1 assay system to address this question. The expression of the *TYB* gene of yeast Ty retrotransposon requires a ribosomal +1 frameshift event caused by a peptidyl-tRNA slippage (41). Only a seven nucleotide sequence CUU-AGG-C is required for the +1 frameshift event to occur and thus only two tRNA species—${\rm tRNA}_{{\rm UAG}}^{{\rm Leu}}$and ${\rm tRNA}_{{\rm CCU}}^{{\rm Arg}}$—are participating in this event. In the yeast strain used, the availability of ${\rm tRNA}_{{\rm CCU}}^{{\rm Arg}}$ is low resulting in a low rate of ribosomal A-site selection, which induces a slippage by ${\rm tRNA}_{{\rm UAG}}^{{\rm Leu}}$ at the CUU P-site codon into the +1 frame (UU-A) (41). Therefore, we decided to use an altered version of the Ty1 +1 frameshift system to study whether or not the +1 frameshifting caused by lack of the mcm^5^ side chain in the *R-luc-F-luc* system, is due to a peptidyl-tRNA slippage. We altered the ‘CUU-AGG-C’ +1 frameshift site by changing the Arg codon (AGG) into either a Lys codon AAA decoded by ${\rm tRNA}_{{\rm mcm}^{\rm 5} {\rm s}^{\rm 2} {\rm UUU}}^{{\rm Lys}}$ or an Arg codon AGA decoded by ${\rm tRNA}_{{\rm mcm}^{\rm 5} {\rm UCU}}^{{\rm Arg}}$ to test whether or not lack of mcm^5^ side chain of these tRNAs induce +1 frameshifting (Table 3). If the hypomodified tRNA is inefficiently accepted to the A-site in an *elp3* mutant, the AAA (Lys) and/or AGA (Arg) test codons will act similarly as codons decoded by the low available ${\rm tRNA}_{{\rm CCU}}^{{\rm Arg}}$ resulting in a slow entry to the A-site by the ternary complex containing the unmodified tRNA. If so, the ${\rm tRNA}_{{\rm UAG}}^{{\rm Leu}}$ in the P-site will slip into the +1 frame (from cognate CUU to non-cognate UU-A). All alterations of the Ty1 sequence were made in the *HIS4A::lacZ* frameshift reporter plasmid (see Materials and Methods) (Figure 4B). The levels of frameshifting were calculated by dividing the β-galactosidase values generated from the test construct with the values from the in-frame control construct. Table 3 shows that for the ‘CUU-AAA-C’ Lys codon test construct, lack of mcm^5^ side group in the mcm^5^s^2^U nucleoside of ${\rm tRNA}_{{\rm mcm}^{\rm 5} {\rm s}^{\rm 2} {\rm UUU}}^{{\rm Lys}}$ resulted in 10-fold increased +1 frameshifting in the *elp3* mutant compared to wild type. In contrast, for the ‘CUU-AGA-C’ Arg codon test construct, lack of mcm^5^ side group in ${\rm tRNA}_{{\rm mcm}^{\rm 5} {\rm UCU}}^{{\rm Arg}}$ did not increase frameshifting in the *elp3* mutant compared to the wild type (Table 3). Thus, similar to the results obtained by the luciferase system lack of the mcm^5^ group of ${\rm tRNA}_{{\rm mcm}^{\rm 5} {\rm s}^{\rm 2} {\rm UUU}}^{{\rm Lys}}$ induced increased +1 frameshifting. Although we observed an increased frameshifting for mcm^5^ deficient ${\rm tRNA}_{{\rm mcm}^{\rm 5} {\rm UCU}}^{{\rm Arg}}$ in the luciferase assay system (Table 2), this was not the case using the Ty1 assay system (Table 3).

**Table 3.** Influence of mcm^5^ side chain on reading frame maintenance based on data using the *HIS4A::Ty1::lacZ* reporter system

	tRNA decoding the codon and Its Gene Copy Number		Normalized Frameshift Rates (%)		
Ty1 Frameshift Site	at the P-site	at the A-site	WT	*elp3Δ*	Frameshift Ratios*elp3Δ*/WT
CUU-AGA-C	${\rm tRNA}_{{\rm UAG}}^{{\rm Leu}}$, (3 ^a^)	${\rm tRNA}_{{\rm mcm}^{\rm 5} {\rm UCU}}^{{\rm Arg}}$, (11 ^a^)	0.142 ± 0.066	0.114 ± 0.020	0.80 ^d^
CUU-AAA-C	${\rm tRNA}_{{\rm UAG}}^{{\rm Leu}}$, (3 ^a^)	${\rm tRNA}_{{\rm mcm}^{\rm 5} {\rm s}^{\rm 2} {\rm UUU}}^{{\rm Lys}}$, (7 ^a^)	0.191 ± 0.092	1.907 ± 0.787	**9.99**^**c**^
CUU-AAA-C	${\rm tRNA}_{{\rm UAG}}^{{\rm Leu}}$, (3 ^a^)	${\rm tRNA}_{{\rm mcm}^{\rm 5} {\rm s}^{\rm 2} {\rm UUU}}^{{\rm Lys}}$, (7 ^a^ +4 ^b^)	0.076 ± 0.013	0.239 ± 0.020	**3.14**^**c**^
AAA-AGG-C	${\rm tRNA}_{{\rm mcm}^{\rm 5} {\rm s}^{\rm 2} {\rm UUU}}^{{\rm Lys}}$, (7 ^a^)	${\rm tRNA}_{{\rm CCU}}^{{\rm Arg}}$, (1 ^a^)	1.203 ± 0.558	0.892 ± 0.225	0.74 ^d^
AAA-CGT-C	${\rm tRNA}_{{\rm mcm}^{\rm 5} {\rm s}^{\rm 2} {\rm UUU}}^{{\rm Lys}}$, (7 ^a^)	${\rm tRNA}_{{\rm ACG}}^{{\rm Arg}}$, (6 ^a^)	0.053 ± 0.021	0.039 ± 0.010	0.74 ^d^
AAA-ATT-C	${\rm tRNA}_{{\rm mcm}^{\rm 5} {\rm s}^{\rm 2} {\rm UUU}}^{{\rm Lys}}$, (7 ^a^)	${\rm tRNA}_{{\rm AAU}}^{{\rm Ile}}$, (13 ^a^)	0.023 ± 0.008	0.025 ± 0.009	1.07 ^d^

^a^Genomic copy numbers of the tRNA genes.

^b^Copy number of pMB38–9merWT/9merFF plasmid carrying the *tK(UUU)L* gene encoding ${\rm tRNA}_{{\rm mcm}^{\rm 5} {\rm s}^{\rm 2} {\rm UUU}}^{{\rm Lys}}$ (41,58).

^c^Difference in frameshifting between *elp3* mutant and wild type was significant as determined by two-tail *t-*test (*P* < 0.02).

^d^Difference in frameshifting between *elp3* mutant and wild type was not significant as determined by two-tail *t-*test (*P* > 0.05).

To analyze whether lack of the mcm^5^ group of ${\rm tRNA}_{{\rm mcm}^{\rm 5} {\rm s}^{\rm 2} {\rm UUU}}^{{\rm Lys}}$ could induce +1 frameshifting due to a P-site effect by modification deficiency, we placed a Lys codon AAA instead of the CUU codon in the Ty1 assay system and varied the following codon. The concentration of a tRNA species is proportional to the number of the corresponding tRNA genes in the yeast genome (50). Accordingly, by placing different codons in the A-site, the concentration of the corresponding tRNAs in the cell reading this codon is changed and thereby the efficiency of reading the A-site codon is altered. Thus, to test for a possible P-site effect induced by a lack of the mcm^5^ group of mcm^5^s^2^U in Lys-tRNA we placed an Arg codon AGG read by the rare cognate ${\rm tRNA}_{{\rm CCU}}^{{\rm Arg}}$ (1 genomic copy) and the near cognate ${\rm tRNA}_{{\rm mcm}^{\rm 5} {\rm UCU}}^{{\rm Arg}}$ (11 genomic copies) after the Lys codon AAA. In an *elp3* mutant ${\rm tRNA}_{{\rm CCU}}^{{\rm Arg}}$ is essential, demonstrating that mcm^5^ group of the near cognate Arg-tRNA is required for efficient reading of the Arg codon AGG (26). Therefore, in an *elp3* mutant, a situation is generated where the AGG codon is read slowly since it is read mainly by the rare cognate ${\rm tRNA}_{{\rm CCU}}^{{\rm Arg}}$ and inefficiently by the more abundant modification deficient near cognate ${\rm tRNA}_{{\rm mcm}^{\rm 5} {\rm UCU}}^{{\rm Arg}}$. Such a condition would allow ${\rm tRNA}_{{\rm mcm}^{\rm 5} {\rm s}^{\rm 2} {\rm UUU}}^{{\rm Lys}}$ at the P-site to slip to the +1 translational frame. Furthermore, we made test constructs to increase the rate of A-site selection by introducing either an Ile codon AUU decoded by cognate ${\rm tRNA}_{{\rm AAU}}^{{\rm Ile}}$ present in 13 genomic copies or an Arg codon CGU decoded by cognate ${\rm tRNA}_{{\rm ACG}}^{{\rm Arg}}$ present in 6 genomic copies after Lys codon AAA (Table 3). By varying concentration of the potential A-site coding tRNAs from 1 genomic copy to 13 genomic copies, we did not observe any significant difference in the levels of +1 frameshifting between wild type and *elp3* mutant (Table 3). Apparently, the possible peptidyl-${\rm tRNA}_{{\rm mcm}^{\rm 5} {\rm s}^{\rm 2} {\rm UUU}}^{{\rm Lys}}$ slippage is not sensitive to the rate of A-site selection suggesting that lack of mcm^5^s^2^U does not cause any P-site effect and thus an increased peptidyl-tRNA slippage.

If the frameshifting event occurring at the modified Ty1 site ‘CUU-AAA-C’ was caused by a slow entry of the ternary complex containing the hypomodified ${\rm tRNA}_{{\rm mcm}^{\rm 5} {\rm s}^{\rm 2} {\rm UUU}}^{{\rm Lys}}$ causing a peptidyl-${\rm tRNA}_{{\rm UAG}}^{{\rm Leu}}$ slippage to +1 translational frame, an elevated level of the hypomodified ${\rm tRNA}_{{\rm mcm}^{\rm 5} {\rm s}^{\rm 2} {\rm UUU}}^{{\rm Lys}}$should increase the rate of A-site selection and thereby reducing +1 frameshifting (Figure 1B). We therefore cloned the *tK(UUU)L* gene, which encodes ${\rm tRNA}_{{\rm mcm}^{\rm 5} {\rm s}^{\rm 2} {\rm UUU}}^{{\rm Lys}}$ into either plasmid pMB38–9merWT (test construct, containing the CUU-AAA-C frameshift site) and pMB38–9merFF (corresponding in-frame control construct, Figure 4 and Supplementary Table S2). Thus, the plasmids harbor both the tRNA gene and the β-galactosidase gene with either a frameshift site or an in-frame control. The plasmid encoded *tK(UUU)L* gene results in overexpression of ${\rm tRNA}_{{\rm mcm}^{\rm 5} {\rm s}^{\rm 2} {\rm UUU}}^{{\rm Lys}}$ and concomitantly reduced the levels of +1 frameshifting in the *elp3* mutant from 10- to 3-fold compared to wild type (Table 3). This data strongly suggest that the +1 frameshifting event at ‘CUU-AAA-C’ Lys codon test construct occurs by peptidyl-${\rm tRNA}_{{\rm UAG}}^{{\rm Leu}}$ slippage due to an A-site effect caused by a slow entry of the hypomodified ${\rm tRNA}_{{\rm mcm}^{\rm 5} {\rm s}^{\rm 2} {\rm UUU}}^{{\rm Lys}}$. As was suggested earlier by us (34,48) and confirmed by Rezgui et al. (51), the major function of the mcm^5^s^2^U_34_ nucleoside in Lys-tRNA is to improve the reading of the cognate codon. Thus, mcm^5^s^2^U_34_ deficiency results in slow decoding and reduced translation elongation rate but also, as shown here, induces +1 frameshifting by reducing the rate of A-site selection.

## CONCLUSION

Among the tRNA isoacceptors having xm^5^U_34_ or xm^5^s^2^U_34_ wobble uridine nucleosides, only Lys- and Gln-tRNAs has been investigated for +1 frameshifting in both bacteria and yeast. The modified wobble nucleoside 5-methoxycarbonylmethyl-2-thiouridine (mcm^5^s^2^U_34_) present in yeast tRNAs specific for Gln, Lys and Glu has a chemically related form, 5-methylaminomethyl-2-thiouridine (mnm^5^s^2^U_34_) present in the corresponding bacterial tRNAs. In bacteria, lack of the mnm^5^ group in Gln-tRNA results in increased +1 frameshifting at both cognate (CAA) and near cognate (CAG) codons, whereas absence of the s^2^ group results in +1 frameshifting only at the cognate (CAA) codon (6). In contrast, lack of mcm^5^ or s^2^ groups in yeast Gln-tRNA does not result in increased +1 frameshifting at either CAA or CAG codons. Instead, absence of the s^2^ group results in reduced +1 frameshifting at the CAG codon. In bacteria, lack of mnm^5^ or s^2^ groups in Lys-tRNA cause increased +1 frameshifting at both cognate (AAA) and near cognate (AAG) codons by A- and P-site effects (6). In yeast, we also observed an increased +1 frameshifting due to lack of mcm^5^ or s^2^ groups of Lys-tRNA at AAA and AAG codons. However, we show that +1 frameshifting at the cognate (AAA) codon is induced by an A-site effect, not a P-site effect.

It has been shown that presence of modified nucleosides in tRNAs are required for tuning the decoding activity in order to maintain uniformity in translation (49). An *in vitro* study in yeast showed that presence of the mcm^5^ and s^2^ groups of Lys-tRNA are required for efficient A-site binding (51). Consistent with these observations, our *in vivo* studies show that presence of the mcm^5^ group of Lys-tRNA promotes its entry to ribosomal A-site and thereby avoids +1 frameshift errors. Thus, wobble uridine modifications are required to optimize the function of tRNAs and thereby promote a proper reading frame maintenance.

## Supplementary Material

SUPPLEMENTARY DATA
