# Supplementary material for: The role of wobble uridine modifications in +1 translational frameshifting in eukaryotes
Source: Nucleic Acids Res. 2015 Aug 17;43(19):9489–99. doi: 10.1093/nar/gkv832 (PMC4627075; doi:10.1093/nar/gkv832)
Supplement: SUPPLEMENTARY DATA [file supp_43_19_9489__index.html]

The role of wobble uridine modifications in +1 translational frameshifting in eukaryotes — The role of wobble uridine modifications in +1 translational frameshifting in eukaryotes — SUPPLEMENTARY DATA 

# The role of wobble uridine modifications in +1 translational frameshifting in eukaryotes

## SUPPLEMENTARY DATA

- SUPPLEMENTARY DATA
