# Supplementary material for: The role of wobble uridine modifications in +1 translational frameshifting in eukaryotes
Source: Nucleic Acids Res. 2015 Aug 17;43(19):9489–99. doi: 10.1093/nar/gkv832 (PMC4627075; doi:10.1093/nar/gkv832)
Supplement: SUPPLEMENTARY DATA [file supp_gkv832_nar-02186-r-2015-File006.pdf]

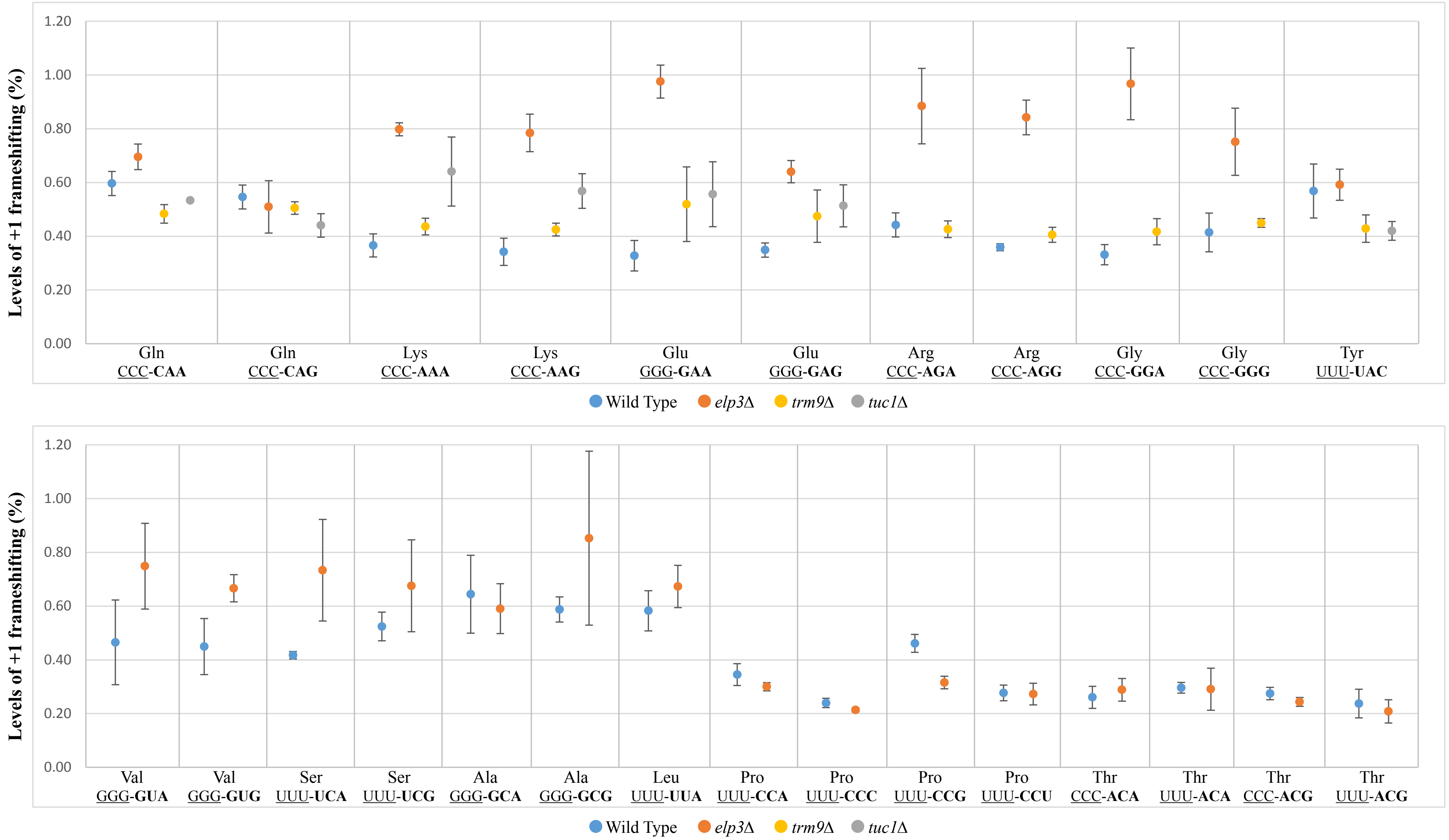

Figure S1. Levels of +1 frameshifting in the constructs with various A- or G- ending test codons decoded by tRNAs lacking the ncm^5 group, the mcm^5 group, the esterified methyl group of mcm^5 or the s^2 group at wobble position. Levels of +1 frameshifting were analyzed by using wild type (blue) and mutant strains (*elp3Δ*: red, *trm9Δ*: yellow, *tuc1Δ*: grey). Each frameshifting value is the mean of three biological replicates and the value of the each biological replicate is the median of three technical replicates. Slippery codons (XXX) are underlined and test codons (**NNN**) are in bold. Error bars were determined by standart deviation.

Table S1. Plasmids used for luciferase assay system.

Plasmids	Fragments inserted between two luciferase genes [a]	tRNA assayed	Source
ABY1830	GGA UCC CCC GGG GAG CUC	In-frame control	(40)
ABY1843	GGA UCC CUA GGC **CCC** **CAA** UAG C GAG CUC	$tRNA^{Gln}_{mcm^5s^2UUG}$	This study
ABY1846	GGA UCC CUA GGC **CCC** **CAG** UAG C GAG CUC		This study
ABY1844	GGA UCC CUA GGC **CCC** **AAA** UAG C GAG CUC	$tRNA^{Lys}_{mcm^5s^2UUU}$	This study
ABY1847	GGA UCC CUA GGC **CCC** **AAG** UAG C GAG CUC		This study
ABY2049	GGA UCC CUA GGC **GGG** **GAA** UAG C GAG CUC	$tRNA^{Glu}_{mcm^5s^2UUC}$	This study
ABY1845	GGA UCC CUA GGC **GGG** **GAG** UAG C GAG CUC		This study
ABY1838	GGA UCC CUA GGC **CCC** **AGA** UAG C GAG CUC	$tRNA^{Arg}_{mcm^5UCU}$	This study
ABY1839	GGA UCC CUA GGC **CCC** **AGG** UAG C GAG CUC		This study
ABY1840	GGA UCC CUA GGC **CCC** **GGA** UAG C GAG CUC	$tRNA^{Gly}_{mcm^5UCC}$	This study
ABY1842	GGA UCC CUA GGC **CCC** **GGG** UAG C GAG CUC		This study
ABY1837	GGA UCC CUA GGC **GGG** **GUA** UAG C GAG CUC	$tRNA^{Val}_{ncm^5UAC}$	This study
ABY2007	GGA UCC CUA GGC **GGG** **GUG** UAG C GAG CUC		This study
ABY2008	GGA UCC CUA GGC **UUU** **UCA** UAG C GAG CUC	$tRNA^{Ser}_{ncm^5UGA}$	This study
ABY2009	GGA UCC CUA GGC **UUU** **UCG** UAG C GAG CUC		This study
ABY1835	GGA UCC CUA GGC **GGG** **GCA** UAG C GAG CUC	$tRNA^{Ala}_{ncm^5UGC}$	This study
ABY1836	GGA UCC CUA GGC **GGG** **GCG** UAG C GAG CUC		This study
ABY2006	GGA UCC CUA GGC **UUU** **UUA** UAG C GAG CUC	$tRNA^{Leu}_{ncm^5UAA}$	This study
ABY1833	GGA UCC CUA GGC **UUU** **CCA** UAG C GAG CUC	$tRNA^{Pro}_{ncm^5UGG}$	This study
ABY1832	GGA UCC CUA GGC **UUU** **CCC** UAG C GAG CUC		This study
ABY1834	GGA UCC CUA GGC **UUU** **CCG** UAG C GAG CUC		This study
ABY1831	GGA UCC CUA GGC **UUU** **CCU** UAG C GAG CUC		This study
ABY2048	GGA UCC CUA GGC **CCC** **ACA** UAG C GAG CUC	$tRNA^{Thr}_{ncm^5UGU}$	This study
ABY2012	GGA UCC CUA GGC **UUU** **ACA** UAG C GAG CUC		This study
ABY2011	GGA UCC CUA GGC **CCC** **ACG** UAG C GAG CUC		This study
ABY2013	GGA UCC CUA GGC **UUU** **ACG** UAG C GAG CUC		This study
ABY2014	GGA UCC CUA GGC **UUU** **UAC** UAG C GAG CUC	$tRNA^{Tyr}_{GUA}$	This study

[a] Restriction sites *Bam*HI-*Sac*I are marked with dotted line. Slippery codons are in bold and test codons are in bold and underlined.

Table S2. Oligonucleotides used to alter the Ty1 sequence of plasmids pMB38-9merWT and pMB38-9merFF.

Plasmids	Template [a]	Oligonucleotides used for PCR directed mutagenesis (5’ to 3’) [b]	tRNA assayed	Source
ABY2138	pMB38-9merWT Test Construct	CCGCTGACA(**CTT)(AGA)C**CATGAGGTACCCG’	$tRNA^{Arg}_{mcm^{5}UCU}$	This study
ABY2139		CTCGACGGATCCGCTGACA(**CTT)(AAA)C**CATGAGGTACC	$tRNA^{Lys}_{mcm^{5}s^{2}UUU}$	This study
ABY2140 [c]		GGCTCGACGGATCCGCTGACA(**AAA)(AGG)**CATGAGG	$tRNA^{Lys}_{mcm^{5}s^{2}UUU}$	This study
ABY2141 [c]		GATGGCTCGACGGATCCGCTGACA(**AAA)(CGT)C**CATGAGGTACCCGA’	$tRNA^{Lys}_{mcm^{5}s^{2}UUU}$	This study
ABY2142 [c]		GGATGGCTCGACGGATCCGCTGACA(**AAA)(ATT)C**CATGAGGTACCCGAT	$tRNA^{Lys}_{mcm^{5}s^{2}UUU}$	This study
ABY2143	pMB38-9merFF In-frame Control Construct	CCGCTGACA(**CTT)(AGA)C**CACGTGAGGTACC	$tRNA^{Arg}_{mcm^{5}UCU}$	This study
ABY2144		CGACGGATCCGCTGACA(**CTT)(AAA)C**CACGTGAGGT	$tRNA^{Lys}_{mcm^{5}s^{2}UUU}$	This study
ABY2145 [c]		GGCTCGACGGATCCGCTGACA(**AAA)(AGG)C**CACGTGA	$tRNA^{Lys}_{mcm^{5}s^{2}UUU}$	This study
ABY2146 [c]		GGATGGCTCGACGGATCCGCTGACA(**AAA)(CGT)C**CACGTGAGGTAC	$tRNA^{Lys}_{mcm^{5}s^{2}UUU}$	This study
ABY2147 [c]		TTGGATGGCTCGACGGATCCGCTGACA(**AAA)(ATT)C**CACGTGAGGTACC	$tRNA^{Lys}_{mcm^{5}s^{2}UUU}$	This study

[a] pMB38-9merWT (test construct) and pMB38-9merFF (in-frame control construct) plasmids carrying the Ty1 sequence (CTT-AGG-C) were used as templates for the PCR mutagenesis (41).
[b] Alterations of the Ty1 sequence are labelled in bold.
[c] Constructs to test the P-site induced frameshifting.
